# Compressed sensing with stochastic spikes

**DOI:** 10.1186/1471-2202-12-S1-P251

**Published:** 2011-07-18

**Authors:** David Rotermund, Klaus R Pawelzik

**Affiliations:** 1 Institute for Theoretical Physics, University of Bremen, Bremen, 28334, Germany

## 

Compressed Sensing (CS) refers to the mathematical finding that perfect reconstruction of a high dimensional state can be possible also from much lower dimensional samples provided the state representation is sufficiently sparse. Since neuronal activities in cortex are in fact sparse it is tempting to explain certain aspects of neuronal coding in terms of CS ([1] and [2]). The applicability of CS to neuronal structures and activities, however, critically relies on realistic assumptions about the neuronal mechanisms that could implement efficient algorithms. Here we investigated the feasibility of CS with rate coding neurons. Also, we are interested in the speed-precision tradeoff of reconstructions using spike-based algorithms similar to the one we introduced previously [3].

We find that a biologically plausible algorithm for non-negative activities can efficiently exploit the information contained in stochastic spike events and converges to close solutions for a wide range of sparsenesses and under-samplings.

Figure 1 shows the root mean squared error (relative to the initial error) averaged over 10 examples depending on proportion of zeros in the representation on the abscissa and the compression ratio M/N on the ordinate, where M is number of observed neurons and N (here set to 500) is the dimension of the underlying state.

**Figure 1:**
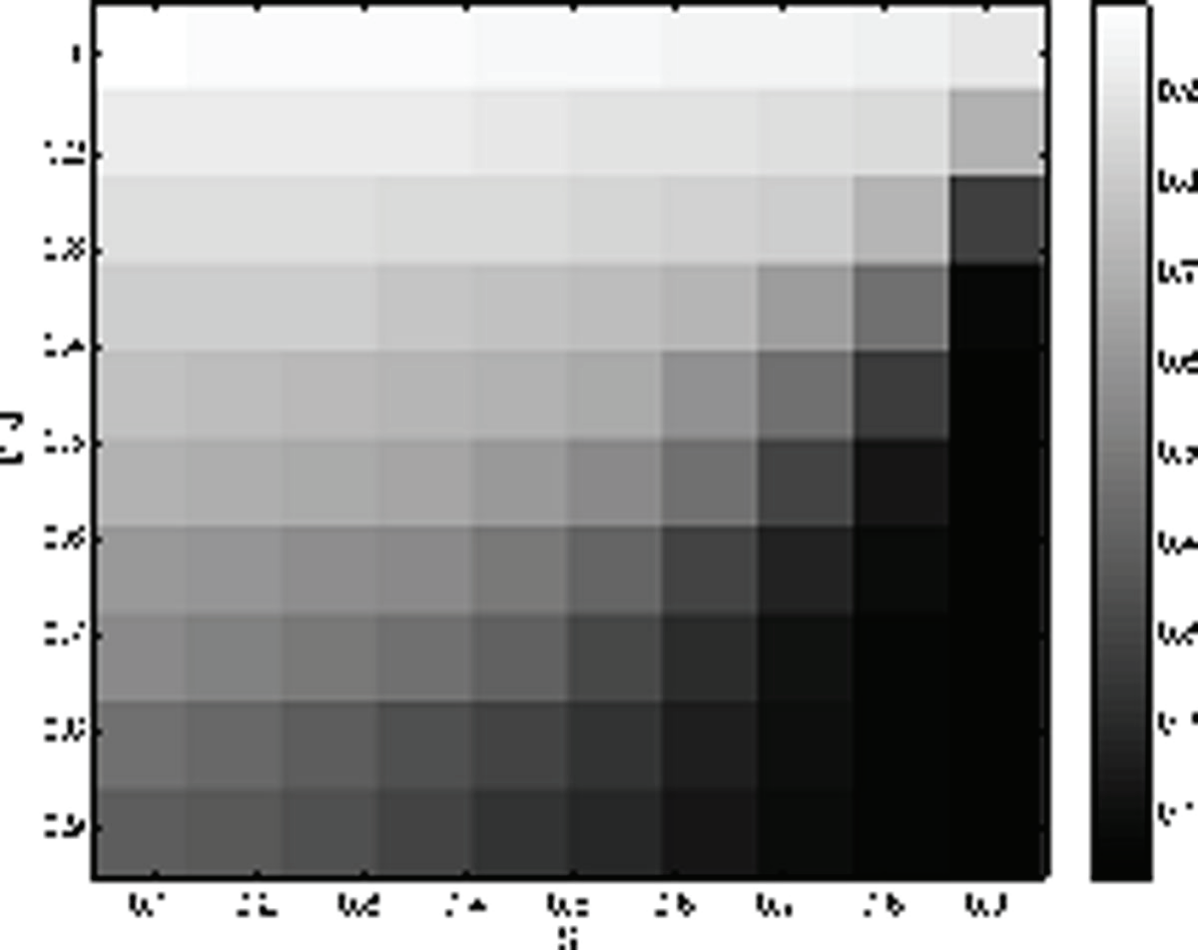


We also investigated conditions on the generating matrix that would facilitate satisfactory reconstructions from limited numbers of spikes. Learning such structures with sparseness constraints can speed up estimations but will in general not match the 'true' generating model. Therefore the construction of sparse representations from spikes can be considered a bias favoring speed in contrast to faithfulness. In [3] we showed that learning generating matrices is possible using only spike activity. Taken together, our results underline the potential relevance of CS for understanding connectivity structures, sparseness and activity dynamics in the brain.

## References

[B1] Olshausen BA Field DJ Emergence of Simple-Cell Receptive Field Properties by Learning a Sparse Code for Natural Images Nature 1996 381 607 609 10.1038/381607a0 8637596

[B2] Coulter WK Hillar CJ Sommer FT Adaptive compressed sensing – a new class of self-organizing coding models for neuroscience NIPS 2010

[B3] Ernst U Rotermund D Pawelzik KR Efficient computation based on stochastic spikes Neural Computation 2007 19 5 1313 1343 10.1162/neco.2007.19.5.1313 17381268

